# Higher ambulatory blood pressure relates to enlarged Virchow-Robin spaces in first-ever lacunar stroke patients

**DOI:** 10.1007/s00415-012-6598-z

**Published:** 2012-07-07

**Authors:** Pim Klarenbeek, Robert J. van Oostenbrugge, Jan Lodder, Rob P. W. Rouhl, Iris L. H. Knottnerus, Julie Staals

**Affiliations:** 1 Department of Neurology, Maastricht University Medical Centre, PO Box 5800, 6202 AZ Maastricht, The Netherlands; 2 Cardiovascular Research Institute Maastricht (CARIM), Maastricht University, Maastricht, The Netherlands

**Keywords:** Enlarged Virchow-Robin spaces, Blood pressure, Ambulatory blood pressure monitoring, Cerebral small vessel disease, Enlarged perivascular spaces

## Abstract

Enlarged Virchow-Robin spaces (EVRS) are considered to be a sign of cerebral small vessel disease. Hypertension is an important risk factor for cerebral small vessel disease, whereas ambulatory blood pressure (BP) is the strongest predictor of hypertension-related brain damage. However, the association between ambulatory BP levels and EVRS has never been investigated. The aim of this study was to determine the association between ambulatory BP levels and EVRS. In 143 first-ever lacunar stroke patients, we performed 24-h ambulatory BP monitoring after the acute stroke phase. On brain MRI we counted EVRS in the basal ganglia and the centrum semiovale. We graded the number of EVRS at each level into a three-category severity scale. We assessed the association between BP levels and EVRS by ordinal regression analysis. After adjusting for age, sex, extensive white matter lesions, and asymptomatic lacunar infarcts, higher day systolic (OR 1.21; 95 % CI 1.00–1.46 per 10 mmHg), day diastolic (1.18; 95 % CI 1.02–1.37 per 5 mmHg) and 24-h diastolic (OR 1.18; 95 % CI 1.01–1.37 per 5 mmHg) ambulatory BP levels were associated with EVRS at the basal ganglia level. No relation was found between ambulatory BP levels and EVRS in the centrum semiovale. Higher day ambulatory BP levels are associated with EVRS in the basal ganglia. This association was independent of the presence of extensive white matter lesions and asymptomatic lacunar infarcts. Our results imply that basal ganglia EVRS should be regarded as a separate manifestation of BP-related brain damage.

## Introduction

Virchow-Robin spaces are perivascular spaces that surround the perforating vessels in the brain. These perivascular spaces are continuous with the perivascular space around the arteries in the subarachnoid space and likely play a role in the drainage of interstitial fluid from the brain [1, 18]. When enlarged, Virchow-Robin spaces can be seen along the course of the perforating vessels on brain MRI as punctuate or linear hyperintensities, isointense to cerebrospinal fluid, in the basal ganglia (BG) or centrum semiovale (CSO) [13]. Enlarged Virchow-Robin spaces (EVRS) are seen with increasing age [10], and are related to worse cognitive functioning, which makes it clinically relevant to recognize them and to search for treatable risk factors [15].

EVRS are considered to be a sign of cerebral small vessel disease since they are associated with the lacunar stoke subtype, white matter lesions and asymptomatic lacunar infarcts [6, 19]. Hypertension is considered an important and modifiable risk factor for cerebral small vessel disease [4, 28]. The positive association of hypertension with (asymptomatic) lacunar infarcts and white matter lesions has been well established [11, 16, 20, 21], but data for EVRS are scarce. A recent study found an association between the degree of EVRS and hypertension, defined by office blood pressure (BP) measurements [31]. However, hypertension is an arbitrarily dichotomized and qualitative term and it would be better to analyze BP as a quantitative, continuous variable. Furthermore, ambulatory BP is a stronger predictor of hypertension-related brain damage than office BP [17, 21]. We aimed to investigate the association between EVRS and ambulatory BP levels in patients with clinical cerebral small vessel disease, i.e., lacunar stroke patients.

## Methods

### Patients

For a lacunar stroke research project, we prospectively recruited patients who presented with a first-ever lacunar stroke at Maastricht University Medical Centre, the Netherlands, from May 2003 to January 2008 and at Orbis Medical Centre Sittard, the Netherlands, from September 2004 to April 2007. The present study was a sub-study of this lacunar stroke research project. As described earlier [23], we defined lacunar stroke as an acute lacunar stroke syndrome with a recent, small, deep infarct on MRI (basal ganglia, internal capsule or brain stem) compatible with the clinical findings. We used established criteria of specific clinical lacunar syndromes if no symptomatic lesion was visible [2]. As we aimed to study patients who most likely had their stroke from small vessel disease, patients with a potential cardioembolic source or carotid artery stenosis >50 % were excluded. We documented the following vascular risk factors: age, sex, diabetes mellitus, current smoking, and hypercholesterolemia (total cholesterol level >5 mmol/l).

### MRI scoring

**Fig. 1:**
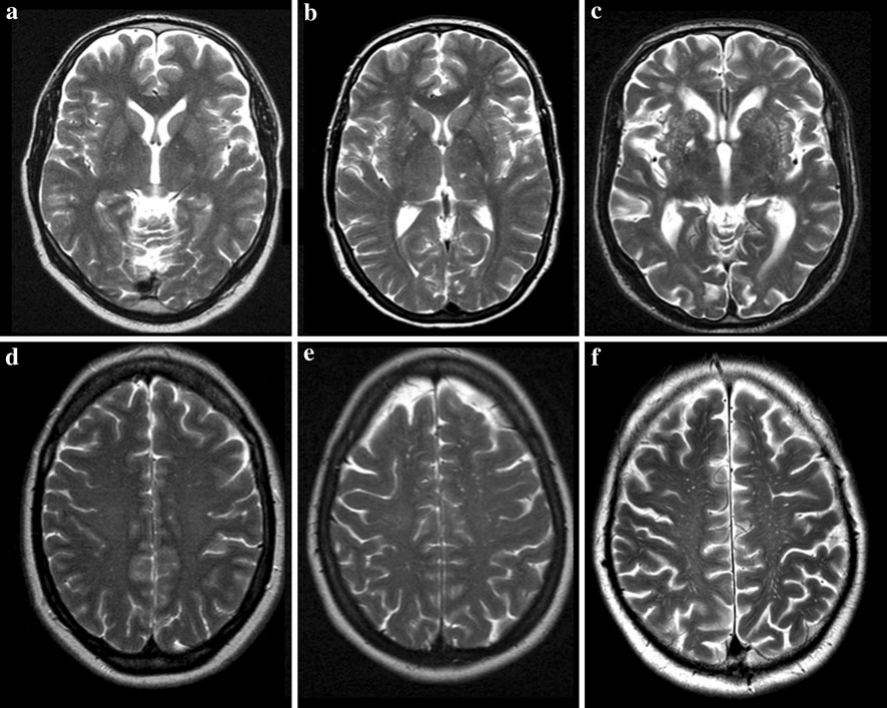
Examples of the three ordinal categories of enlarged Virchow-Robin spaces. **a**–**c** Enlarged Virchow-Robin spaces (EVRS) in the basal ganglia, 0–10 EVRS (category 1); 10–25 EVRS (category 2); and >25 EVRS (category 3), respectively. **d**–**f** EVRS in the centrum semiovale, 0–10 EVRS (category 1); 10-25 EVRS (category 2); and >25 EVRS (category 3), respectively

MR images (1.5-T, Philips, the Netherlands) were obtained within 6 months after stroke (mean 40.6 ± 42.6 days). We performed standard axial T2-weighted fast spin echo (TR shortest, TE 100 ms; field of view 230 mm; matrix 512 × 512) and axial fluid attenuated inversion recovery (FLAIR) (TR 8,000 ms, TE 120 ms; inversion time 2,000 ms; field of view 230 mm; matrix 256 × 256 reconstructed to 512 × 512) images, all with slice thickness of 5 mm and gaps of 0.50 mm. We defined EVRS as round, oval, or linear-shaped structures that follow the orientation of the perforating vessels with a diameter <3 mm, a smooth margin, and absence of mass effect. On T2-weighted MR images, EVRS have a signal intensity equal to cerebrospinal fluid and (if visible) they appear hypointense on FLAIR images without a hyperintense rim to distinguish them from cavitated lacunar infarcts [3, 25]. We distinguished EVRS at two different levels: the BG and the CSO. This distinction was made because these two levels represent different vascular territories with a possible different pathogenesis of EVRS. At both levels, we identified the slide with the highest number of EVRS in one hemisphere. We then graded the number of EVRS in a three-category ordinal scale (Fig. 1) as follows: 0–10 EVRS (category 1); 10–25 EVRS (category 2); and >25 EVRS (category 3). Our scoring method was adapted from recently published studies investigating EVRS with slight modifications [6, 15]. EVRS were independently rated by two vascular neurologists (interobserver agreement testing on all scans was fair to good; weighted Cohen’s kappa 0.73 for EVRS in the BG and 0.71 for EVRS in the CSO) [9]. If the rating differed, the EVRS category was determined by consensus. All MR images were graded for asymptomatic lacunar infarcts as well as white matter lesions. Asymptomatic lacunar infarcts were defined as hyperintense lesions on T2-weighted images with corresponding hypointense lesions with a hyperintense rim on FLAIR in the BG, internal capsule, or brainstem, diameter <20 mm, and not compatible with clinical findings. White matter lesions were graded according to the Fazekas scale from 0 to 3 [7, 8].

### BP measurements

Ambulatory BP monitoring (using Mobil O Graph equipment, IEM GbmH, Stolberg, Germany) was performed after the acute stroke phase, between 1 and 6 months post-stroke (mean 101.2 ± 42.3 days). Measurements over a 24-h period were obtained every 15 min during the day (07:00–23:00 hours) and every 30 min during the night (23:00–7:00 hours). Patients continued their prescribed medication, and we registered the use of antihypertensive drugs. Patients kept a record of rising and retiring times [23]. We determined day and night periods by excluding a 2-h transition period around the reported rising and retiring times. Measurement data were not edited manually. Valid recordings required a minimum of 15 daytime and eight night-time measurements. We calculated mean 24-h, day, and night systolic and diastolic BP.

### Statistical analysis

Statistical analysis was performed using SPSS (version 16.0 for Windows, SPSS Inc., Chicago, IL, USA). Data are presented as *n* (%), as mean ± SD for parametric data, or as median (range) for nonparametric data. Differences between groups were determined using Chi-square (*χ* ^2^) test or one-way ANOVA where appropriate. We assessed the relationship between the various BP levels and EVRS by ordinal regression analysis adjusting for age and sex (model 1). We tested whether age was an effect modifier of the association between BP and EVRS by adding the relevant interaction term into the model. As *p* values of the interaction term for each of the BP variables were all >0.05, we further omitted the term. We then adjusted for the presence of asymptomatic lacunar infarcts and extensive white matter lesions (model 2). Extensive white matter lesions were defined as (early) confluent deep white matter hyperintensities (Fazekas score 2 and 3) and/or irregular periventricular hyperintensities extending into the deep white matter (Fazekas score 3) on FLAIR and T2-weighted images. We used these Fazekas scores because they are histopathologically related to cerebral small vessel disease [8]. Finally, additional exploratory analysis was performed by adding one-by-one the vascular risk factors and the number of antihypertensive drugs used by each patient to model 2. Statistical significance was considered at *p* < 0.05.

## Results

### Patient characteristics

Of 281 first-ever lacunar stroke patients at Maastricht University Medical Centre, 35 were excluded because of carotid artery stenosis or possible cardioembolic source (most commonly atrial fibrillation), 116 refused to participate or had contra-indications for MRI and 16 were excluded because of inadequate MRI or ambulatory BP monitoring data, leaving 114 patients for the present study. Included subjects were younger (65.3 years ± 11.5 vs. 71.6 years ± 12.0, *p* < 0.001) and more often male (62.3 vs. 45.8 %, *p* < 0.01). By applying the same criteria, we recruited 29 patients from Orbis Medical Centre Sittard (number and characteristics of non-included patients were not listed), which totals 143 patients.

Table 1 shows the characteristics for all included subjects. Table 2 presents the relevant characteristics for the different subgroups based on EVRS score at the BG and CSO. Higher numbers of EVRS at the BG related to increasing age as well as to the presence of asymptomatic LACI and extensive WML. The documented vascular risk factors did not differ between groups and are not shown. EVRS in the BG and CSO were correlated at Spearman rho 0.37 (*p* < 0.001).

**Table 1:** Patient characteristics

Characteristic	All patients (*n* = 143)
Age (years)	63.0 ± 12.2
Male sex	87 (60.8 %)
Diabetes mellitus	18 (12.6 %)
Hypercholesterolemia	115 (80.4 %)
Current smoking	57 (40.0 %)
Extensive white matter lesions	49 (34.3 %)^a^
Asymptomatic lacunar infarcts	91 (63.3 %)
Use of antihypertensive drugs	89 (62.2 %)
Number of drugs used	1 (0–5)
24 h SBP (mmHg)	139 ± 17.5
24 h DBP (mmHg)	83 ± 12
Day SBP (mmHg)	144 ± 18
Day DBP (mmHg)	86 ± 13
Night SBP (mmHg)	125 ± 19
Night DBP (mmHg)	73 ± 12

Absolute numbers with percentages between* brackets*; mean ± SD median with range

*SBP* systolic blood pressure, *DBP* diastolic blood pressure

^a^Deep WML Fazekas’ scores 0–3 were 42 (29.4 %), 59 (41.3 %), 17 (11.9 %) and 25 (17.5 %) respectively; periventricular WML Fazekas’ scores 0–3 were 56 (39.2 %), 41 (28.7 %), 8 (5.6 %) and 38 (26.6 %), respectively

**Table 2:** Characteristics of subgroups based on EVRS score

	EVRS in BG			EVRS in CSO		
	Cat. 1 (*n* = 49)	Cat. 2 (*n* = 55)	Cat. 3 (*n* = 39)	Cat. 1 (*n* = 30)	Cat. 2 (*n* = 65)	Cat. 3 (*n* = 48)
Age	55 ± 10*	65 ± 12*	71 ± 10*	62 ± 13*	61 ± 12*	67 ± 11*
Male (%)	30 (61)	34 (62)	23 (59)	19 (63)	38 (59)	30 (63)
eWML (%)	7 (14)^†^	18 (33)^†^	24 (62)^†^	11 (37)	22 (34)	16 (33)
aLACI (%)	21 (43)^†^	37 (67)^†^	33 (85)^†^	20 (67)	36 (55)	35 (73)
24 h SBP	135 ± 17	143 ± 19	140 ± 15	137 ± 19	139 ± 18	141 ± 16
24 h DBP	82 ± 12	85 ± 14	82 ± 8	82 ± 13	84 ± 13	82 ± 9
Day SBP	139 ± 17	147 ± 20	144 ± 15	141 ± 19	144 ± 19	145 ± 16
Day DBP	85 ± 12	88 ± 15	85 ± 8	84 ± 14	87 ± 14	85 ± 10
Night SBP	120 ± 18	129 ± 21	127 ± 16	123 ± 19	124 ± 20	129 ± 18
Night DBP	72 ± 12	75 ± 13	73 ± 9	72 ± 13	74 ± 13	73 ± 10

Absolute numbers with percentages between* brackets*; mean ± SD

* p < 0.01; ^†^ *p* < 0.001

*EVRS* enlarged Virchow-Robin spaces, *BG* basal ganglia, *CSO* centrum semiovale, *SBP* systolic blood pressure, *DBP* diastolic blood pressure, *eWML* extensive white matter lesions, *aLACI* asymptomatic lacunar infarcts

### BP levels and EVRS in the BG

Ambulatory BP levels for the three EVRS categories at the BG are presented in Table 2. Table 3 shows the results of the ordinal regression analyses. After adjustment for age and sex, higher 24-h and day systolic and diastolic BP were associated with higher numbers of EVRS. With additional adjustment for asymptomatic lacunar infarcts and extensive white matter lesions higher day systolic and diastolic BP and 24-h diastolic BP remained significantly associated with the number of EVRS. Exploratory analysis with additional adjustment for any one of the vascular risk factors (diabetes mellitus, current smoking, and hypercholesterolemia) or number of antihypertensive drugs used by each patient did not change the results substantially.

**Table 3:** Ambulatory BP characteristics in relation to Basal ganglia EVRS

Mean BP (mmHg)	Unadjusted OR (95 % CI)	Model 1 (95 % CI)	Model 2 (95 % CI)
24 h			
SBP	1.15 (0.97−1.38)	1.21 (1.00–1.45)*	1.19 (0.97–1.43)
DBP	1.01 (0.89–1.14)	1.22 (1.05–1.43)^†^	1.18 (1.01–1.37)*
Day			
SBP	1.14 (0.96–1.35)	1.22 (1.02–1.46)*	1.21 (1.00–1.46)*
DBP	1.00 (0.89–1.13)	1.22 (1.06–1.41)^†^	1.18 (1.02–1.37)*
Night			
SBP	1.14 (0.97–1.34)	1.11 (0.93–1.31)	1.06 (0.89–1.27)
DBP	1.04 (0.91-1.17)	1.14 (1.00–1.32)	1.08 (0.94−1.25)

Results of ordinal regression analyses presented as OR per 10 mmHg increase in systolic BP or 5 mmHg in DBP. Model 1 adjusted for age and sex. Model 2 additionally adjusted for extensive white matter lesions and asymptomatic lacunar infarcts

*BP* blood pressure, *EVRS* enlarged Virchow-Robins spaces, *SBP* systolic blood pressure, *DBP* diastolic blood pressure

* p < 0.05; ^†^ *p* < 0.01

### BP levels and EVRS in the CSO

Ambulatory BP levels for the three EVRS categories at the CSO are also presented in Table 2. There was no association of ambulatory BP levels with the number of EVRS in the CSO (Table 4).

**Table 4:** Ambulatory BP characteristics in relation to centrum semiovale EVRS

Mean BP (mmHg)	Unadjusted OR (95 % CI)	Model 1 (95 % CI)	Model 2 (95 % CI)
24 h			
SBP	1.11 (0.92–1.32)	1.12 (0.93–1.32)	1.09 (0.91–1.31)
DBP	1.00 (0.88–1.13)	1.05 (0.91–1.20)	1.04 (0.90–1.20)
Day			
SBP	1.08 (0.91–1.28)	1.09 (0.92–1.30)	1.08 (0.90–1.28)
DBP	1.00 (0.88–1.12)	1.05 (0.92–1.20)	1.04 (0.91–1.20)
Night			
SBP	1.13 (0.96–1.32)	1.11 (0.94–1.31)	1.09 (0.92–1.30)
DBP	1.02 (0.89–1.16)	1.03 (0.90–1.19)	1.03 (0.90–1.18)

Results of ordinal regression analyses presented as OR per 10 mmHg increase in systolic BP or 5 mmHg in DBP. Model 1 adjusted for age and sex. Model 2 additionally adjusted for extensive white matter lesions and asymptomatic lacunar infarcts

*BP* blood pressure, *EVRS* enlarged Virchow-Robins spaces, *SBP* systolic blood pressure, *DBP* diastolic blood pressure

## Discussion

We studied the association between BP analyzed as a continuous variable, using ambulatory BP monitoring, and EVRS. We found that higher day systolic, day diastolic, and 24-h diastolic ambulatory BP levels are associated with higher numbers of EVRS in the BG in first-ever lacunar stroke patients. This association was independent of age, sex, the presence of extensive white matter lesions, and the presence of asymptomatic lacunar infarcts.

Recently Zhu et al. found an association between the severity of EVRS (in both the BG and CSO) and hypertension in a large population-based cohort of elderly individuals [31]. Previously, two other studies investigated the association between EVRS and hypertension. In one retrospective study, the association between EVRS and hypertension disappeared after correction for age, sex, dementia, and white matter lesions [10]. In the other study, an association between linear EVRS in the CSO and hypertension was shown in a small cohort of 26 patients (EVRS in the BG were not counted in this study) [12]. All three studies used office BP measurements to define hypertension, which is inferior to ambulatory BP monitoring in predicting hypertension-related brain damage [17, 21, 24].

Similar to other studies [6, 19, 31], we found that increasing numbers of EVRS in the BG were positively associated with extensive white matter lesions and asymptomatic lacunar infarcts. Because there is a known association between higher blood pressure levels and extensive white matter lesions and asymptomatic lacunar infarcts, one could dismiss any relation between higher blood pressure levels and EVRS as only a derivate of the former association. However, although the relationship between EVRS and higher BP levels weakened somewhat after correction for white matter lesions and asymptomatic lacunar infarcts in our cohort, it remained significant for 24-h diastolic BP and day systolic and diastolic BP. This implies that EVRS are a distinct manifestation of BP-related brain damage and this supports the hypothesis that EVRS are a separate manifestation of cerebral small vessel disease as has been suggested in earlier studies [6, 19].

Elevated BP causes endothelial dysfunction [5, 27]. Recent evidence suggests that increased blood–brain barrier permeability (i.e., endothelial dysfunction) is involved in the pathogenesis of cerebral small vessel disease [30]. This increased blood–brain barrier permeability causes extravasation of fluid and blood products into the vessel wall and perivascular space, with the appearance of EVRS on MRI [14, 29]. The association of ambulatory BP levels with EVRS in the BG but not in the CSO is in line with previous reports [6, 19, 31]. This could be explained by the fact that these are different vascular territories with differences in the vulnerability of the vessel wall to the influence of BP, however this remains hypothetical.

Most ambulatory BP-monitoring studies report an association between white matter lesions and/or lacunar infarcts and day as well as night BP [21, 22, 26]. In contrast to these studies, our results show an association of EVRS in the BG with day but not night BP. Our current study does not provide an explanation for this. Longitudinal studies will provide better insight, as they will measure the effect of different BP levels on the progression of the different MRI markers of cerebral small vessel disease over time.

The main strength of our study is that we used ambulatory BP monitoring instead of office BP measurements. Furthermore, we collected a homogeneous group of lacunar stroke patients, for which there was no other cause than cerebral small vessel disease. In this group, EVRS are more prevalent than in the general population, which makes this group very suitable for studying the association between BP and EVRS. However, our study also has some limitations. First, we found a linear relation of higher age and higher numbers of EVRS. After correction for age, the associations of BP levels and EVRS were significant, indicating that the association is not completely determined by an age effect. However, it is impossible to completely cancel out other age-related effects on BP. Second, our patient selection favors younger, less disabled patients, able to undergo MRI. However, this selection bias would probably lead to an underestimation of the association between EVRS and BP levels. Third, this is a cross-sectional study. Results of ambulatory BP monitoring represent the actual BP level without accounting for BP level, course, duration, and treatment in the past. Besides, we cannot account for a possible effect of the symptomatic lacunar infarct on BP levels, although we performed ambulatory BP monitoring after the acute stroke phase. Longitudinal studies are needed to investigate the progression of EVRS in relation to BP levels. Fourth, we did not use diffusion-weighted imaging to confirm the acute lacunar infarct, and therefore we cannot exclude that we erroneously classified an asymptomatic lacunar lesion as the symptomatic one. However, because all patients had a distinct clinical lacunar stroke syndrome, we feel that this did not lead to unrightfully included patients.

In conclusion, we found an independent association between higher day systolic and diastolic BP levels and the number of EVRS in the BG. Our results imply that EVRS are a separate manifestation of BP-induced brain damage and support the hypothesis that EVRS in the BG should be regarded as a separate manifestation of cerebral small vessel disease.
